# Improvement of an interobserver agreement of ARDS diagnosis by adding additional imaging and a confidence scale

**DOI:** 10.3389/fmed.2022.950827

**Published:** 2022-08-31

**Authors:** Laura A. Hagens, Fleur L. I. M. Van der Ven, Nanon F. L. Heijnen, Marry R. Smit, Hester A. Gietema, Suzanne C. Gerretsen, Marcus J. Schultz, Dennis C. J. J. Bergmans, Ronny M. Schnabel, Lieuwe D. J. Bos

**Affiliations:** ^1^Department of Intensive Care, Amsterdam University Medical Center, Location Amsterdam Medical Center, University of Amsterdam, Amsterdam, Netherlands; ^2^Department of Intensive Care, Rode Kruis Ziekenhuis, Brandwondencentrum, Beverwijk, Netherlands; ^3^Department of Intensive Care, Maastricht University Medical Centre+, Maastricht, Netherlands; ^4^Department of Radiology and Nuclear Medicine, Maastricht University Medical Centre+, Maastricht, Netherlands; ^5^GROW School for Oncology and Reproduction, Maastricht University, Maastricht, Netherlands; ^6^Mahidol-Oxford Tropical Medicine Research Unit (MORU), Mahidol University, Bangkok, Thailand; ^7^Nuffield Department of Medicine, University of Oxford, Oxford, United Kingdom; ^8^Medical Affairs, Hamilton Medical AG, Bonaduz, Switzerland; ^9^School of Nutrition and Translational Research in Metabolism (NUTRIM), Maastricht University, Maastricht, Netherlands; ^10^Department of Respiratory Medicine, Amsterdam UMC, Location AMC, University of Amsterdam, Amsterdam, Netherlands

**Keywords:** ARDS, imaging, CT, chest X-ray, confidence, diagnosis

## Abstract

Acute respiratory distress syndrome (ARDS) often is not recognized in clinical practice, largely due to variation in the interpretation of chest x-ray (CXR) leading to poor interobserver reliability. We hypothesized that the agreement in the interpretation of chest imaging for the diagnosis of ARDS in invasively ventilated intensive care unit patients between experts improves when using an 8-grade confidence scale compared to using a dichotomous assessment and that the agreement increases after adding chest computed tomography (CT) or lung ultrasound (LUS) to CXR. Three experts scored ARDS according to the Berlin definition based on case records from an observational cohort study using a dichotomous assessment and an 8-grade confidence scale. The intraclass correlation (ICC), imaging modality, and the scoring method were calculated per day and compared using bootstrapping. A consensus judgement on the presence of ARDS was based on the combined confidence grades of the experts, followed by a consensus meeting for conflicting scores. In total, 401 patients were included in the analysis. The best ICC was found using an 8-grade confidence scale for LUS (ICC: 0.49; 95%-CI: 0.29–0.63) and CT evaluation (ICC: 0.49; 95%-CI: 0.34–0.61). The ICC of CXR increased by 0.022 and of CT by 0.065 when 8-grade scoring was used instead of the dichotomous assessment. Adding information from LUS or chest CT increased the ICC by 0.25 when using the 8-grade confidence assessment. An agreement on the diagnosis of ARDS can increase substantially by adapting the scoring system from a dichotomous assessment to an 8-grade confidence scale and by adding additional imaging modalities such as LUS or chest CT. This suggests that a simple assessment of the diagnosis of ARDS with a chart review by one assessor is insufficient to define ARDS in future studies.

**Clinical trial registration:**

Trialregister.nl (identifier NL8226).

## Introduction

Acute respiratory distress syndrome (ARDS) is the most common cause for acute hypoxemic respiratory failure in patients requiring admission to the intensive care unit (ICU) (1). However, clinicians often do not recognize ARDS (2). Using the 2012 Berlin definition of ARDS, a previous study attempted to improve the diagnostic validity and reliability of the included criteria using clinical case vignettes (3). Yet, the diagnosis remains challenging, which negatively influences the inclusion criteria in clinical trials and may influence treatment decisions (1). A more reliable recognition of ARDS would facilitate better inclusion in intervention studies and improve the classification of ARDS as reference standard in diagnostic studies (4).

The diagnosis of ARDS remains subjective largely due to variation in the interpretation of chest imaging results (4). The interobserver reliability on the agreement of bilateral opacities on chest x-ray (CXR) is low (4–7). Several studies focused on improving the reliability of CXR interpretation, for example by providing clinical vignettes for training, but these studies had conflicting results (7, 8). Two approaches that seem to improve agreement are the aggregation of multiple images and scoring by multiple experts (4, 5). The inclusion of the rater's confidence in the correct diagnosis rather than the dichotomized judgements can increase the uniformity between raters and may facilitate a more consistent diagnosis (4). Combining CXR with other available lung imaging techniques such as chest CT and lung ultrasound (LUS) may also provide a more reliable assessment. An approach that combines multiple imaging modalities with the confidence scoring method has not been studied in the diagnosis of ARDS.

We hypothesized that the agreement in the interpretation of lung images for the diagnosis of ARDS in invasively ventilated ICU patients is better between experts when using an 8-grade confidence scale than when using a dichotomous assessment and that the agreement increases after adding chest CT or LUS to CXR. Secondly, we developed a method that identified cases with high agreement, taking the confidence of the experts' evaluation into consideration, and we hypothesized that such consensus expert classification has better face validity than the judgement of the treating physician, the team of researchers, or any expert alone.

## Methods

### Design and ethics

This is a pre-planned analysis of data collected in the Diagnosis of Acute Respiratory disTress Syndrome (DARTS) project, which is a prospective multicentre cohort study conducted in the Netherlands. Consecutive patients with an expected duration of invasive ventilation of at least 24 h who were admitted to the ICUs of the Amsterdam University Medical Center (AUMC), location AMC and the Maastricht University Medical Center+ (MUMC+) from March 2019 to February 2021 were included in the study (9). The institutional review board of both centers approved the study protocol (W18_311 #18.358 and 2019-1137). Details on the informed consent procedure are described in a previous study (9).

### Patients

Patients were included for the current analysis if they met the following criteria: PaO_2_/FiO_2_ ≤300 mmHg, positive end expiratory pressure (PEEP) ≥5 cmH_2_O, and availability of at least one chest x-ray or chest CT. There were no exclusion criteria.

### Data collection

Clinical data were collected prospectively in a secured, online database (Castor EDC, Amsterdam, Netherlands) by a team of 3 dedicated clinical researchers (9). Data was collected on the 1st and 2nd day of invasive ventilation.

CXR, LUS, and CT data were collected systematically (9). CXRs were selected from the 1st day and the 2nd day of invasive ventilation, with a maximum delay of 72 h after the initiation of invasive ventilation (CXR1 and CXR2). Chest CT scans were collected within the timeframe of 72 h before intubation until 72 h after intubation.

### Scoring of ARDS

**Figure 1:**
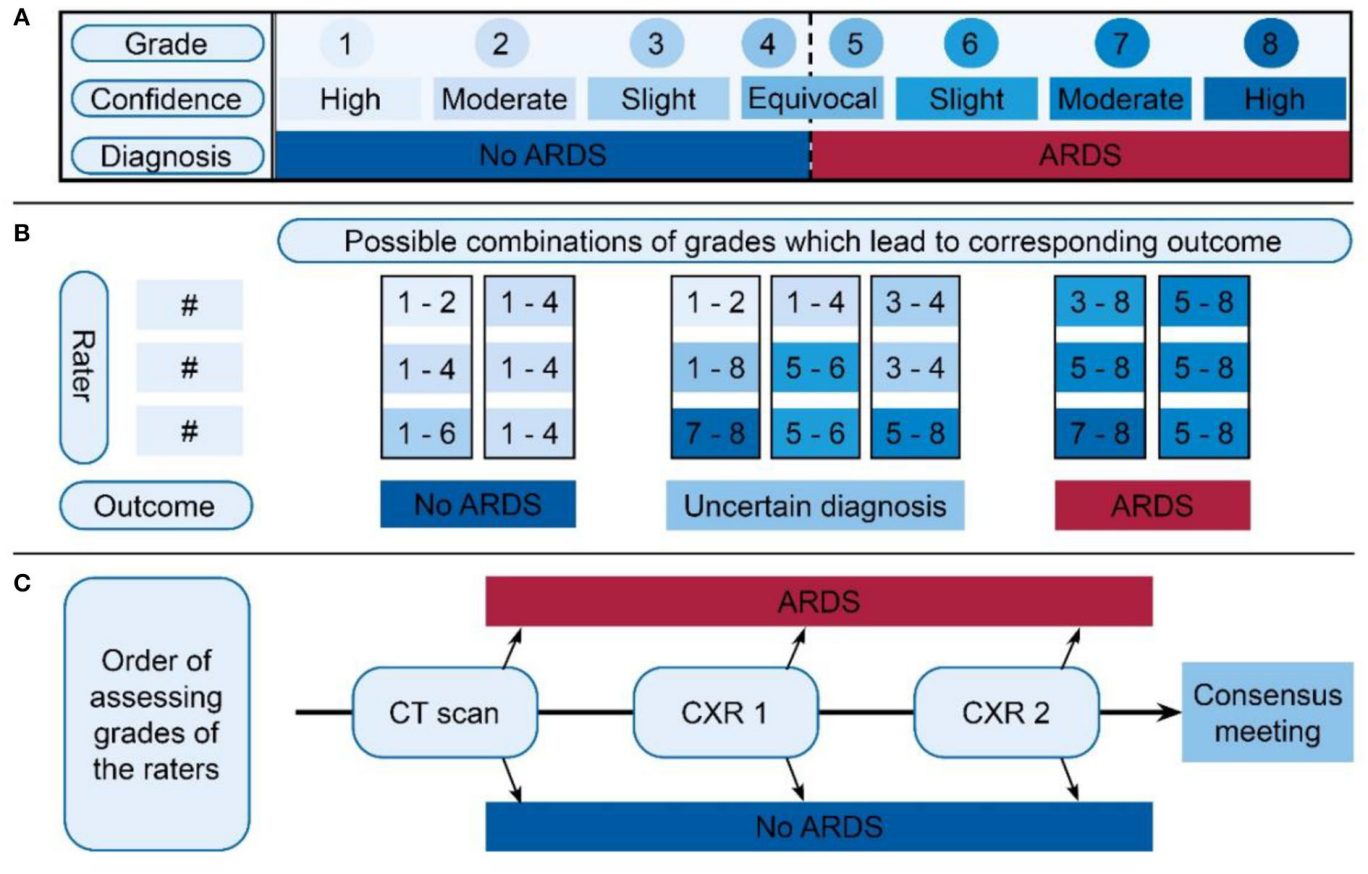
Overview of ARDS scoring and classification, divided into three parts. **(A)** The 8-grade confidence scale on which the expert scored a certain grade for each available image. The upper row displays the grades the expert scored, where the middle row indicates how the confidence of this grade should be interpreted. The row below indicates the corresponding diagnosis. **(B)** Schedule for indexing final classification of ARDS diagnosis. Based on the three grades of the expert panel (three times a value between 1 and 8), a subject was classified in one of the three outcomes: “No ARDS,” “Uncertain diagnosis,” or “ARDS.” The option bars above the outcome indicate the grades given by the experts. The figure shows possible combinations between the three raters in the vertical direction. The boxes show the values that were allowed to come to a score of ARDS or no ARDS. When there was uncertainty between the raters or highly confident conflicting scores, an uncertain classification was given. **(C)** Order of assessing the available grades. For the first CT, if a definitive outcome was available, this was regarded as the final decision. If no was CT available or there was no definitive outcome, the grades of CXR1 were assessed. If this did not give a definitive outcome, the second CXR grades were assessed. Also, if no definitive outcome was available, one should proceed in the direction of the arrow, ending up in the “consensus meeting.” Importantly, this was not the order in which the images were scored by the experts. ARDS, acute respiratory distress syndrome; CXR, chest x-ray.

A panel of three clinical experts with years of experience independently reviewed the available clinical variables and imaging to diagnose ARDS according to the Berlin definition, following a predetermined schedule (Supplementary Figure 1). The available imaging was assessed in the following order: (1) images from day 1, (2) images from day 2, and (3) the chest CT. Each expert scored the CXR and chest CT on (1) the presence of bilateral opacities consistent with ARDS (dichotomous classification used in most studies), (2) the most likely etiology of the opacities (infiltrates, effusions, collapse, or nodules), and (3) the presence of cardiogenic edema, which is the most likely explanation for the abnormalities (10). Thereafter, the same experts decided, based on all available information, whether the patient fulfilled the criteria of the Berlin definition for ARDS. The confidence of this diagnosis was scored on an 8-grade scale (Figure 1A), leading to a grade 1 to 8 corresponding to high confidence of “ARDS” or high confidence of “no ARDS” on both extremes. Scores between 1 and 4 were consistent with not scoring ARDS in dichotomous classification while scores between 5 and 8 were consistent with scoring ARDS.

To evaluate if the treating clinicians had made the diagnosis of ARDS, a full chart review was performed. Furthermore, a team of three clinical researchers evaluated the presence of ARDS prospectively using all available clinical information and imaging data.

### Classification of ARDS based on judgement of the expert panel

Depending on the available imaging, three expert judgements for the confidence grades in ARDS diagnosis for a maximum of five situations (CXR1, LUS1, CXR2, LUS2, and CT) were obtained. An algorithmic evaluation (Figure 1B) of the confidence grades of each expert, per imaging modality per day of evaluation resulted in the classification into three categories: (1) “No ARDS,” if sufficient confidence was reached to exclude ARDS, (2) “ARDS,” if sufficient confidence was reached to diagnose ARDS, and (3) “uncertain diagnosis,” when there was general uncertainty (none of the experts had strong confidence) or conflicting results (some scored ARDS with certainty while others were certain that ARDS was not present). For example, if one expert scored a 7 (moderate certainty ARDS) and two experts a 5 (equivocal ARDS), the patient would be classified as having ARDS (Figure 1B: first column with outcome ARDS).

To reach a final diagnosis, we prioritized imaging with the most accurate information available for classification (Figure 1C). When a CT scan was available, the classification based on the expert's judgement from this CT scan was used. If the score for the CT led to the outcome “ARDS” or “No ARDS,” this was used as the final classification. When CT scan was unavailable or had an “uncertainty diagnosis,” classification based on CXR1 was used. In case the evaluations of CXR1 did not result in a classification of “ARDS” or “No ARDS,” the classification of CXR2 was used, if available. If expert panel assessment of none of these images resulted in a clear diagnosis of ARDS, a consensus meeting was held to determine the final classification for this patient. During this consensus meeting, the three experts reviewed all available data again and decided how to classify the patient resulting in two additional categories: “likely ARDS” and “likely no ARDS.”

The LUS data were not used to classify patients as having ARDS/no ARDS because we wanted to evaluate LUS as an index test and not as a reference test.

### Endpoints

The primary endpoint of this study was the increase in the strength of agreement between experts for the diagnosis of ARDS when going from: (A) dichotomous assessment of bilateral opacities (i.e., yes/no) to an 8-grade confidence scale and (B) assessment of a single CXR to the addition of LUS and CT. The agreement was quantified using the intra class correlation coefficient (ICC).

Secondary endpoints were 3-folded. First, the diagnostic characteristics for the diagnosis of ARDS by a single expert, stratified per imaging modality, were evaluated by comparing them with those of the diagnosis by an expert panel, as the reference standard. Second, the diagnostic characteristics of diagnosis of ARDS by the treating physician and a team of researchers were compared to those of the diagnosis by the expert panel. Third, the face validity was tested by comparing PaO_2_/FiO_2_ and PEEP between patients with no ARDS, likely no ARDS, likely ARDS, and ARDS.

### Sample size calculation

The sample size needed to detect an increase in agreement was calculated based on the known ICCs for the scoring of CXR for the presence of bilateral opacities consistent with ARDS diagnosis (4–7). A relatively optimistic estimate for the ICC for dichotomous scoring of CXR is 0.5 (4). Using the R-package *ICC.Sample.Size*, we calculated that ~234 subjects were needed for a very conservative improvement in ICC from 0.5 to 0.6, using a power of 80% (11).

### Statistical analysis

All statistical analyses were performed in R v4.0.3 (www.r-project.org) using the Rstudio interface. For the primary endpoint, the ICC was calculated based on a two-way random model, based on a single measurement, to assess the agreement. To qualify the agreement, the following values were used for the ICC: perfect: 0.8–1.0, substantial: 0.6–0.8, moderate: 0.4–0.6, fair: 0.2–0.4, and poor: 0–0.2 (6). Confidence intervals (CI) were obtained by bootstrapping. Cohen's Kappa was also calculated for dichotomous scoring methods.

For the secondary endpoints, 2x2 tables were created using the expert panel definition of ARDS as the reference standard. Sensitivity, specificity, the positive-predictive value, and the negative-predictive value were calculated.

## Results

### Patients

From the 519 patients included in the DARTS project, 401 patients were eligible for this study. Of the 118 patients not eligible, 26 had missing chest imaging, 80 had a PaO_2_/FiO_2_ above 300 mmHg and 26 had PEEP below 5 cmH_2_O (Supplementary Figure 2). Table 1 shows the subject characteristics.

**Table 1:** Patient characteristics.

	**All**	**No ARDS**	**ARDS**	***P*-value**
***N***	**401**	**216**	**185**	
**Patient characteristics**				
Age, years *mean (SD)*	63 (13)	63 (14)	63 (13)	0.99
Gender = Man	277 (69)	149 (69)	128 (69)	1
BMI	27.0 [23.7, 30.3]	26.9 [23.3, 30.2]	27.0 [24.2, 30.3]	0.31
**Admission characteristics**			
**Admission type**				<0.001
Emergency surgical	53 (13.2)	38 (17.6)	15 (8.1)	
Medical	297 (74.1)	140 (64.8)	157 (84.9)	
Planned surgical	51 (12.7)	38 (17.6)	13 (7.0)	
**ARDS severity**				
Mild	24 (6.0)	NA	24 (13.0)	
Moderate	97 (24.2)	NA	97 (52.4)	
Severe	64 (16.0)	NA	64 (34.6)	
COVID-19	63 (15.7)	1 (0.5)	62 (33.5)	<0.001
Apache II	20 [15, 25]	20 [16, 26]	20 [15, 24]	0.029
SOFA	9 [7, 11]	10 [8, 12]	9 [7, 11]	0.003
LIPS	5.5 [4.0, 7.0]	4.5 [3.0, 7.0]	6.0 [4.5, 7.5]	<0.001
**Ventilation and gas exchange**			
MV duration at day 1, h	21 [14, 28]	21 [14, 28]	21 [12, 28]	0.65
Compliance, mL/cmH_2_O	32.9 [23.8, 47.3]	35.1 [25.2, 50.0]	29.1 [21.8, 42.4]	0.007
PaO_2_/FiO_2_, mmHg	149 [103, 223]	193 [133, 251]	118 [86, 158]	<0.001
PEEP, cmH_2_O	8 [6, 10]	8 [5, 8]	10 [8, 12]	<0.001
**Imaging**				
CXR1 available	346 (86.3)	188 (87.0)	158 (85.4)	0.74
CXR2 available	103 (25.7)	56 (25.9)	47 (25.4)	0.99
CT available	223 (55.6)	111 (51.4)	112 (60.5)	0.082
**Outcomes**				
Hospital LOS, days	19 [10, 32]	20 [9, 35]	19 [12, 31]	0.46
ICU LOS, days	7.5 [4.0, 15.0]	7.0 [3.0, 13.0]	9.0 [4.0, 16.0]	0.015
ICU mortality	131 (32.7)	63 (29.2)	68 (36.8)	0.21
30 d mortality	152 (37.9)	77 (35.6)	75 (40.5)	0.44

Data presented as no. (%) or median with an interquartile range unless indicated otherwise. P-values were calculated using the chi-square, the T-test, or the Mann-Whitney U-test depending on the type and distribution of the variable. The PaO_2_/FiO_2_ is defined as the lowest PaO_2_/FiO_2_ at 24 h before day 1. Additional characteristics are presented in Supplementary Table 4.

ARDS, acute respiratory distress syndrome; BMI, body mass index; APACHE II, acute physiology and chronic health evaluation II; LIPS, lung injury prediction score; SOFA, sequential organ failure assessment; ICU, intensive care unit; LOS, length of stay; MV, mechanical ventilation; NA, not applicable; PEEP, positive end-expiratory pressure; FiO_2_, fraction of inspired oxygen; PaO_2_, partial pressure of oxygen.

### Confidence scoring

**Figure 2:**
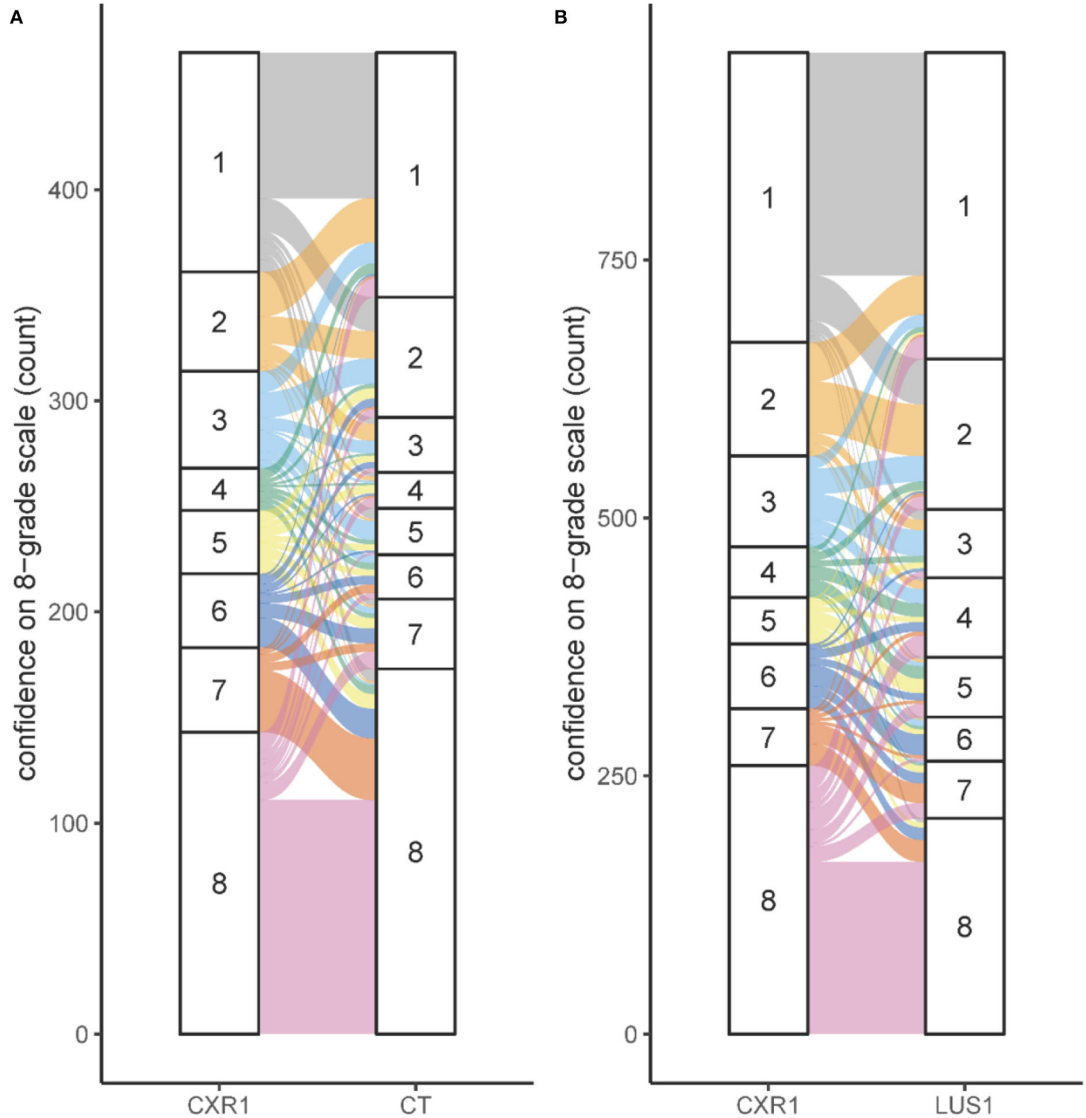
Confidence for several imaging modalities. **(A)** Shift in scoring on the 8-grade confidence scale for the situation CXR1 to CT. **(B)** Shift in scoring on the 8-grade confidence scale for the situation CXR1 to LUS1. The confidence on the 8-grade scale corresponds to the confidence of the ARDS diagnosis, ranging from high confidence no ARDS (grade 1) to high confidence ARDS on both extremes (grade 8), see Figure 1A.

More than 90% of the available images were evaluated based on dichotomous and 8-grade confidence scoring. When an expert scored the CXR with moderate or high confidence (scoring a 1, 2, 7, or 8), this judgement was maintained when reviewing the CT images of that patient (297/334, 89%, Figure 2). However, when the initial confidence was slight or equivocal (scoring a 3, 4, 5, or 6), the CT images changed the confidence scoring toward moderate or high confidence in 82 out of 131 reviews (63%, Figure 2). The addition of LUS did not increase confidence but led to considerable changes in confidence scores (Figure 2B).

### Agreement on scoring ARDS

**Figure 3:**
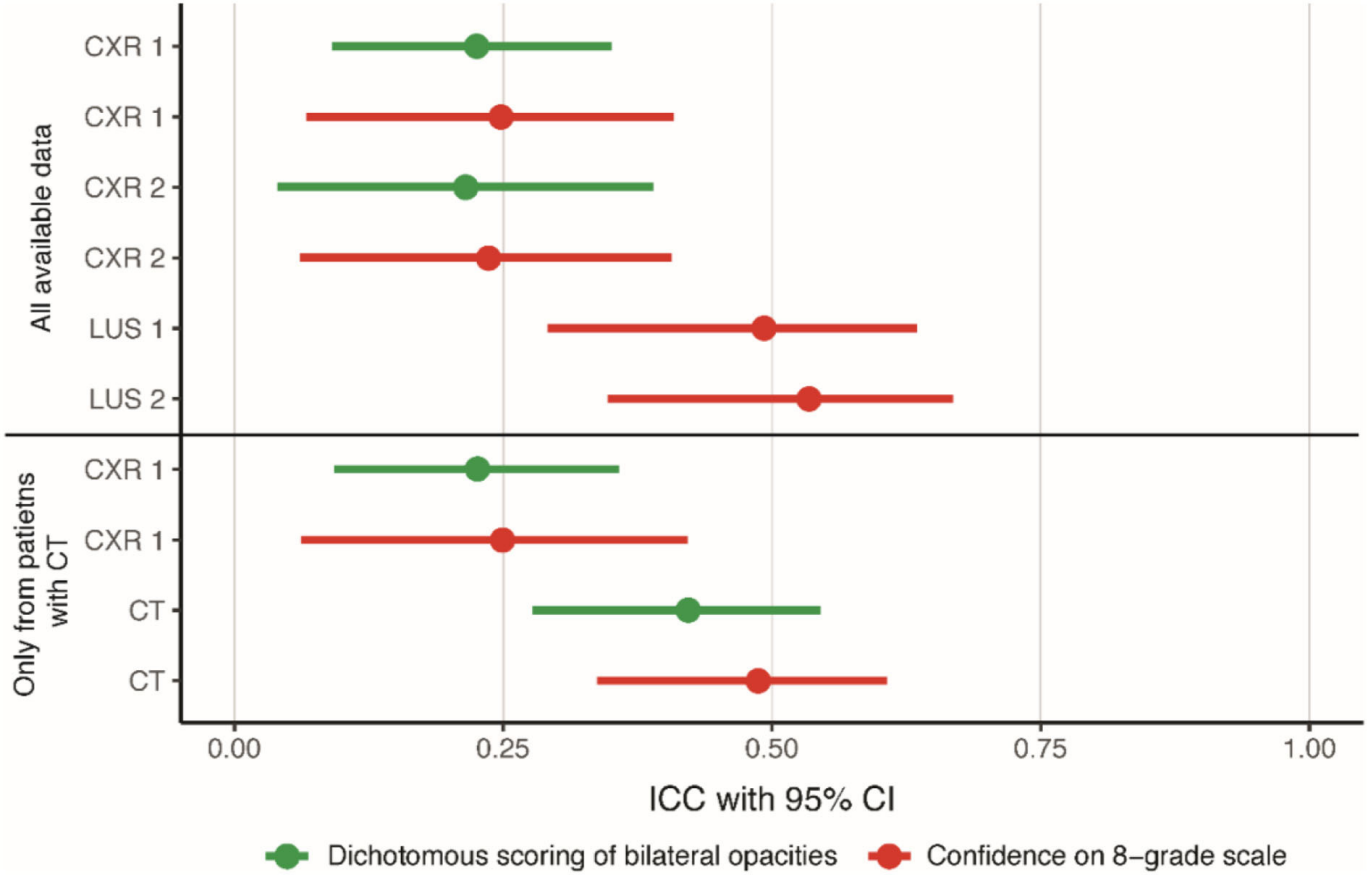
Agreement on scoring ARDS for all imaging modalities. ICCs with a 95% confidence interval displaying the agreement for dichotomous scoring of bilateral opacities to diagnose ARDS and for scoring of confidence for ARDS diagnosis on an 8-grade scale. The confidence on the 8-grade scale corresponds to the confidence of the ARDS diagnosis, ranging from high confidence no ARDS (grade 1) to high confidence ARDS on both extremes (grade 8), see Figure 1A. CXR, chest x-ray; ICC, intra class correlation coefficient; LUS, lung ultrasound.

The agreement on the classification of ARDS depended on the scoring method (dichotomous vs. 8-grade confidence scale) and imaging modality (Figure 3). Dichotomous scoring of bilateral opacities on a CXR showed fair agreement (ICC for CXR1 was 0.23 with a 95% CI of 0.09–0.35 and ICC for CXR2 was 0.21 with a 95% CI of 0.04–0.39). When dichotomous scoring was performed based on a CT scan, agreement improved to moderate (ICC: 0.42; 95%-CI: 0.28–0.52). Similar values were found with Cohen's kappa (CXR1: 0.24, CXR2: 0.23, CT: 0.42). The agreement on the diagnosis of ARDS based on the 8-grade confidence scale ranged from fair based on CXR1 (ICC: 0.25; 95%-CI: 0.07–0.41) to moderate based on LUS1 (ICC: 0.49; 95%-CI: 0.29–0.63) and CT (ICC: 0.49; 95%-CI: 0.34–0.61). There was no change in agreement between day 1 and 2 (Figure 3). Similar results were found when limiting the analysis to patients in whom a chest CT was available (Figure 3; Supplementary Table 1), suggesting that the higher ICC observed for CT images was not due to patient selection.

There was a consistent increase in ICC when moving from a dichotomous assessment to the 8-grade confidence scale (Supplementary Table 2). For CXR1, there was an increase in ICC of 0.022 (95%-CI: 0.020–0.023), and for CXR2, there was an increase of 0.065 (95%-CI: 0.063–0.067). The addition of LUS or CT to CXR also improved the ICC consistently. The scoring of the confidence scale with the addition of LUS increased the ICC by 0.25 (95%-CI: 0.25–0.25) on day 1 and by 0.20 (95% CI: 0.19–0.20) on day 2 compared to the CXR. The CT also increased the agreement, for both scoring methods, with an increase in ICC of 0.20 (95%-CI: 0.20–0.21) for the dichotomous assessment and with an increase in ICC of 0.25 (95%-CI: 0.24–0.25) for the confidence assessment compared to the CXR (Supplementary Table 3).

### Consensus classification of ARDS

The classification of ARDS was based on the confidences scores given by the experts according to the predefined algorithm (Figure 1B). Based on agreement between the three experts, 112 (28%) patients were classified as having ARDS and 137 (34%) patients as having no ARDS. For 152 patients, the diagnosis was uncertain based on the scores from the experts, and a consensus meeting was held. Of these patients, 73 (48%) were classified as likely ARDS and 79 (52%) as likely no ARDS. For the categorization based on final classification, subject characteristics are provided in Supplementary Table 4.

### Diagnostic accuracy for scoring ARDS

The diagnostic accuracy for diagnosing ARDS for the individual experts ranged from 72 to 86% compared to their combined assessment used for the final classification of patients. The research team had an accuracy of 80%, while the clinical team had an accuracy of 57%. All diagnostic test characteristics are listed in Supplementary Table 5.

### Consistency across the categories

**Figure 4:**
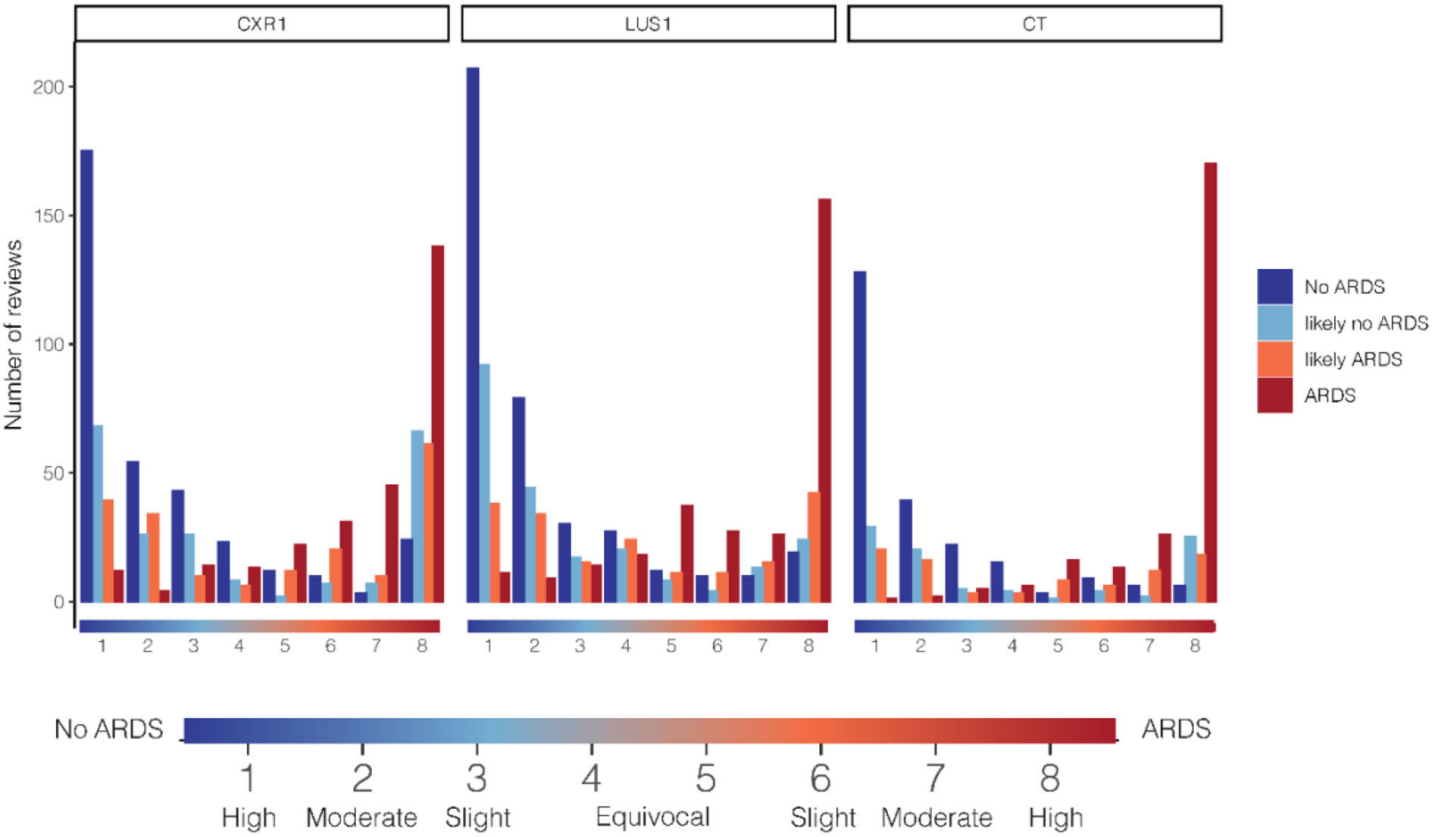
Relationship between cumulative counts per confidence grade and ARDS category in the diagnosis of ARDS. On the x-axis, the confidence score on the 8-grade confidence scale. The confidence on the 8-grade scale corresponds to the confidence of the ARDS diagnosis, ranging from high confidence no ARDS (grade 1) to high confidence ARDS on both extremes (grade 8), see indicator below graph. Cumulative review counts: CXR1: 1,025, LUS1: 1,104, CT: 643. ARDS, acute respiratory distress syndrome; CXR, chest x-ray; LUS, lung ultrasound.

Figure 4 shows the distribution of confidence scores, stratified for imaging modality and grouped by final ARDS classification. Patients with a final diagnosis of ARDS were rarely classified into a certain no ARDS category by one of the experts, while they were more frequently in agreement, showing high confidence or slight confidence in the diagnosis. There was more alignment between the final ARDS classification and the individual experts' confidence judgments for the CT scan than for the CXR.

### Face validity

**Figure 5:**
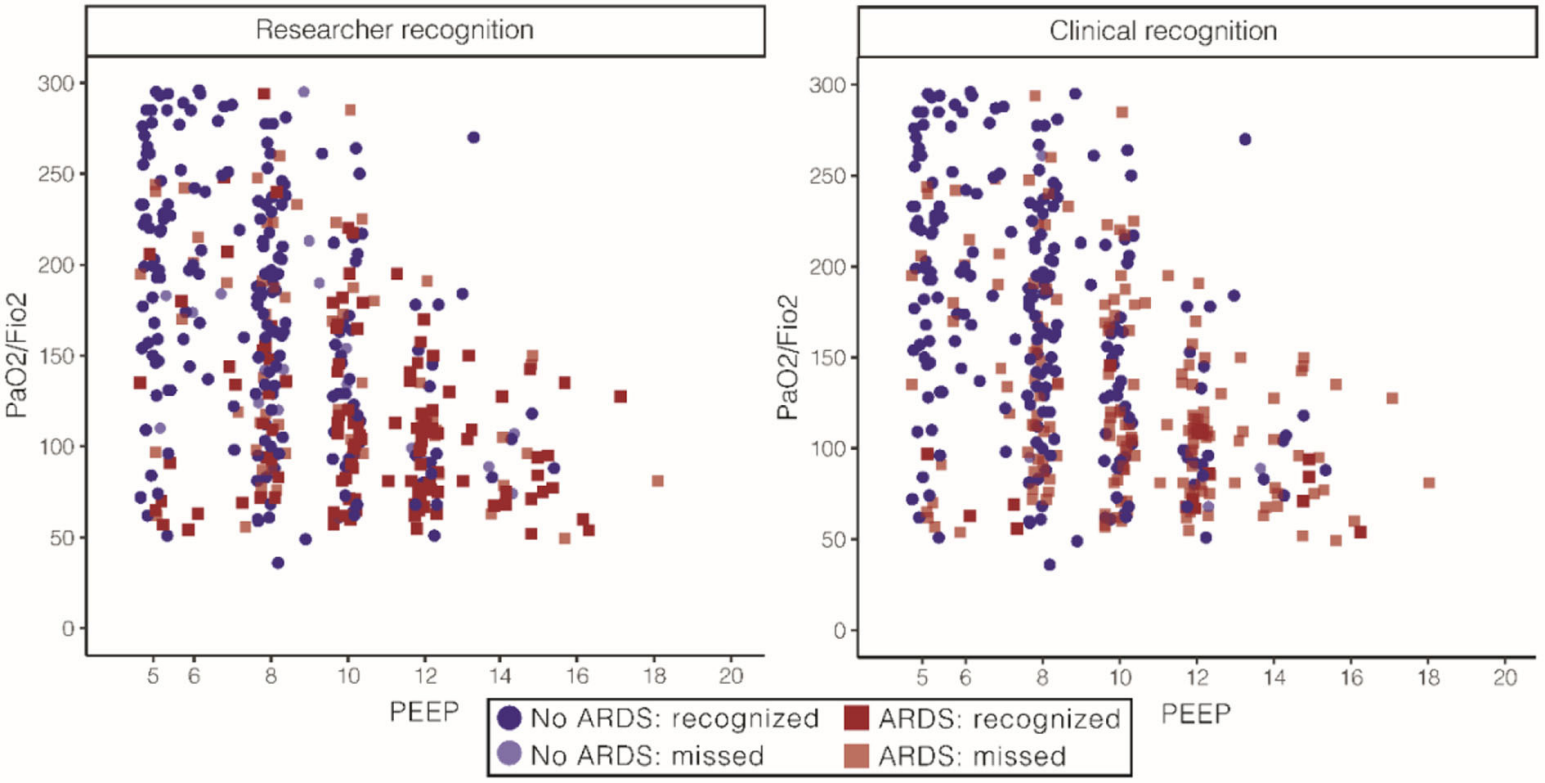
Face validity of ARDS for the expert panel, stratified for researchers and clinical diagnoses. On the left, patient characteristics as recognized by the research team. The research team recognized 66% of the patients classified as ARDS by the expert panel. PaO_2_/FiO_2_ 108 [77, 143] in the ARDS group and 167 [109, 223] the in no ARDS group and PEEP of 10 (8, 12) in the ARDS group and 8 (6, 10) in the no ARDS group [numbers represent median (IQR)]. On the right, patient characteristics as recognized by the clinical team. The clinical team recognized 86% of the patients classified as ARDS by the expert panel. PaO_2_/FiO_2_ 85 [68, 100] in the ARDS group and 144 [98, 201] in the no ARDS group and PEEP of 12 (8, 13) in the ARDS group and 8 (7, 10) in the no ARDS group [numbers represent median (IQR)]. ARDS, acute respiratory distress syndrome; FiO_2_, fraction of inspired oxygen; PaO_2_, partial pressure of oxygen; PEEP, positive end-expiratory pressure.

All patients with ARDS identified by the expert panel, research team, and clinical team had lower PaO_2_/FiO_2_ and higher PEEP than patients who were classified as not having ARDS. Figure 5 illustrates that a larger portion of the included population was recognized as having ARDS by the expert panel than by the research team or clinical team while retaining face validity. The research team recognized 85% of the patients to belonging to the category ARDS, and 37% of the patients were classified as likely ARDS. Only 8% of the patients that were recognized as having ARDS by the research team did not have ARDS according to the expert panel. The clinical team recognized a small minority of patients with ARDS: only 11% in the ARDS category and 6% in the category likely ARDS.

## Discussion

This study showed that the agreement on diagnosis of ARDS can increase substantially by adapting the scoring system from dichotomous to an 8-grade confidence scale and by adding additional imaging modalities like LUS or chest CT. Based on an algorithmic evaluation of these 8-grade confidence ratings between three experts, patients were frequently classified as having ARDS while maintaining the clinical characteristics associated with ARDS indicating face validity. The team of treating physicians did not recognize ARDS in the majority of cases.

Our study confirmed that ARDS diagnosis based on reading of the chest x-ray alone has a fair agreement. This result is comparable to other studies where the reliability was found to be poor (4, 5). Other studies attempted to increase the reliability by providing additional training, but this approach failed (5, 7). This can be explained by the poor capturing technique of chest x-rays in invasively ventilated patients, which are made in a supine position and from anterior to posterior position without deep inspiration, combined with a two-dimensional assessment of the three-dimensional structure that the lung is, which causes over projection of structures. Taken together, it is questionable if a dichotomous assessment of chest x-rays is suitable for ARDS diagnosis.

In this study, we confirm that the use of the confidence scale in the assessment of ARDS diagnosis on an 8-grade scale results in more reliable results, although the improvement in ICC was relatively small (4). Given the difficulties with interpreting chest x-rays, there are many patients in whom a rater may feel uncertain. When this is ignored through dichotomania (12), a patient with a probability of having ARDS of 51% according to the rater is lumped together with a patient in whom the rater reaches 99% certainty. The presented data indeed show that low confidence in the diagnosis is common and that disagreement is more common in these patients.

We also established that the addition of LUS or chest CT further improves inter-rater reliability. LUS provides the possibility to reconstruct loss of aeration at, or directly beneath the pleural surface of the lung and is likely complementary to chest x-ray, which is less suited for the evaluation of subpleural consolidation (13). Therefore, it may not be surprising that the combination of both techniques improves agreement. On the other hand, there is no uniform definition for ARDS based on LUS patterns (14, 15), which could have caused an increased variation between raters. LUS reached the same concordance as chest CT, but the former led to a more certain ARDS diagnosis. CT therefore remains the best imaging modality for ARDS diagnosis, while LUS is a promising modality to study further. An important next step is to establish evidence based diagnostic criteria for the diagnosis of ARDS based on LUS.

The main strength of this study was that we combined several imaging modalities and scoring methods to improve the reliability of ARDS diagnosis using data from a large multicentre study. Experts were blinded for the researcher, the researcher's clinical diagnosis, and each other's assessments, which likely limits bias. A limitation of this study was that the chest imaging was presented in a fixed order so that the rater always reviewed the chest x-ray before evaluating additional imaging techniques. We therefore did not evaluate what the independent value of LUS for ARDS diagnosis is. Simultaneously our approach mimics the most common clinical situation, where a chest x-ray is available routinely and additional imaging in the form of LUS or CT can be added. A second potential limitation is that the experts only had access to a curated set of clinical characteristics in order to keep them blinded. Although this included all information required for scoring ARDS according to the Berlin definition, it may have missed some nuances that are only available when evaluating the patient's bedside. We also only used the judgement of three experts in the judgement of the images, so one could argue that the results of these experts are not directly applicable to other experts. On the contrary, similar disagreements between experts have been found in other studies, and the included experts are clinicians who routinely screen and include patients into ARDS trials, suggesting that they are a representative sample of the target population and might even have more experience in diagnosing ARDS than most others.

The major implication of this study is that the diagnosis of ARDS based on a single clinical evaluation of the chest x-ray is evidently unreliable, such that it is an inadequate reference standard for diagnostic studies and will likely result in arbitrary decisions toward inclusion or exclusion into clinical trials. We therefore suggest adding either CT imaging or LUS to chest x-ray evaluation for ARDS diagnosis when possible as this will increase the reliability of the assessment considerably. Furthermore, when studying a diagnostic test for ARDS, it is likely necessary for the available imaging to be evaluated by at least three experts using an 8-grade confidence scale and to use the hereby presented diagnostic algorithm to assign patients with an ARDS diagnosis. The scoring of the confidence scale rather than the dichotomous assessment hardly costs additional time but provides additional accuracy.

## Conclusion

In this cohort, we found an increase in the strength of the agreement between experts for the diagnosis of ARDS when going from a dichotomous assessment to the 8-grade confidence scale and when adding additional imaging like LUS and chest CT in invasively ventilated ICU patients.

## Data availability statement

The raw data supporting the conclusions of this article will be made available by the authors, without undue reservation.

## Ethics statement

The studies involving human participants were reviewed and approved by Amsterdam UMC: Location AMC. The patients/participants provided their written informed consent to participate in this study.

## Members of the DARTS consortium

Amsterdam UMC: Laura A. Hagens, Marry R. Smit, Marcus J. Schultz, Lieuwe D.J. Bos.

Maastricht UMC: Nanon F.L. Heijnen, Dennis C.J.J. Bergmans, Ronny M. Schnabel.

Philips Research: Alwin R.M. Verschueren, Tamara M.E. Nijsen, and Inge Geven.

## Author contributions

LH and LB had full access to all the data in the study, takes full responsibility for the integrity of the data, accuracy of the data analysis, designed the study, and prepared the initial draft of this manuscript. NH, MRS, MJS, DB, and RS advised on the study design and participated in the study protocol. LH, FV, NH, MRS, HG, SG, RS, and LB participated in data collection. The analysis was performed by LH. All authors approved the submitted version of this manuscript.

## Funding

This work was supported by a grant from Health Holland through the Longfonds awarded to the DARTS consortium.

## Conflict of interest

Author MJS was employed by Medical Affairs, Hamilton Medical AG. Author LB is a consultant for Sobi and Scailyte, which is paid to the institution, and received grants from Longfonds, Health Holland, IMI, and Amsterdam UMC, which is paid to the institution. The remaining authors declare that the research was conducted in the absence of any commercial or financial relationships that could be construed as a potential conflict of interest. The handling editor DB declared a past co-authorship with the author MJS.

## Publisher's note

All claims expressed in this article are solely those of the authors and do not necessarily represent those of their affiliated organizations, or those of the publisher, the editors and the reviewers. Any product that may be evaluated in this article, or claim that may be made by its manufacturer, is not guaranteed or endorsed by the publisher.

## Abbreviations

APACHE II: acute physiology and chronic health evaluation II
ARDS: acute respiratory distress syndrome
BMI: body mass index
CI: confidence interval
CT: computed tomography
CXR: chest x-ray
FiO_2_: fraction of inspired oxygen
ICC: intraclass correlation coefficient
ICU: intensive care unit
LIPS: lung injury prediction score
LOS: length of stay
LUS: lung ultrasound
MV: mechanical ventilation
NA: not applicable
PaO_2_: partial pressure of oxygen
PEEP: positive end-expiratory pressure
SOFA: sequential organ failure assessment.
