# Supplementary material for: Improvement of an interobserver agreement of ARDS diagnosis by adding additional imaging and a confidence scale
Source: Front Med (Lausanne). 2022 Aug 31;9:950827. doi: 10.3389/fmed.2022.950827 (PMC9473335; doi:10.3389/fmed.2022.950827)
Supplement: Supplementary file 1 [file Data_Sheet_1.PDF]

Online supplement

Improvement of interobserver agreement of ARDS diagnosis by adding additional imaging and a confidence scale

Laura A. Hagens [1], Fleur L.I.M. Van der Ven [1,3], Nanon F.L. Heijnen [4], Marry R. Smit [1], Hester A. Gietema [5, 10], Suzanne C. Gerretsen [5], Marcus J. Schultz [1,6,7,8], Dennis C.J.J. Bergmans 4,9], Ronny M. Schnabel [4], Lieuwe D.J. Bos [1,2]

On behalf of the DARTS consortium.

Content:

Supplemental methods

Supplemental results

Supplemental figures: E-Figure 1 to E-Figure 2

Supplemental tables: E-Table 1 to E-Table 5

Methods

LUS examination

LUS was performed as part of the study at the first and second day of invasive ventilation according to the 12-region protocol (LUS1 & LUS2). LUS was performed with the clinically available ultrasound device using a linear transducer according to a previously published protocol [14]. To ascertain good inter-rater reliability, all researchers were trained by experienced physicians in LUS examination beforehand. Previous research has shown that this leads to an excellent inter-rater variability with an ICC of 0.98 [16]. Six examination regions were selected per hemi thorax. Anterior axillary line and posterior axillary line divide the chest into anterior, lateral and posterior [17]. Each chest zone was scanned, the most pathologic finding was saved. Scoring was performed according to the regional aeration score; A (presence of lung sliding and A-lines), B1 (presence of >2 separated B-lines covering less than 50% of the pleura), B2 (presence of B-lines that cover more than 50% of the pleura) or C (presence of consolidative lung tissue with diameter >2 cm) [18]. Also the presence of subpleural consolidations was scored.

Results

ARDS scoring

On day 1, 342/342 (100%) CXRs and 350 LUS examinations were scored by all three experts. On day 2, 99/100 (99%) CXRs and 193 LUS examinations were scored by all three experts. A total of 202/221 (91%) CTs was scored completely.

E-Figures

E-Figure 1: Study procedures in assessment of patients

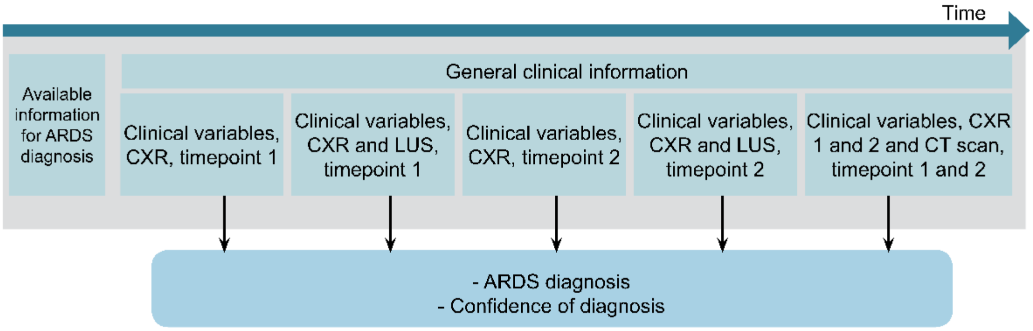

Expert assesses the available imaging and scores on each arrow if a patient full fills the criteria of the Berlin Definition.

Abbreviations: ARDS: Acute Respiratory Distress Syndrome, CT = computed tomography, CXR: chest X-ray, LUS: lung ultrasound.

E-Figure 2: Inclusion flow

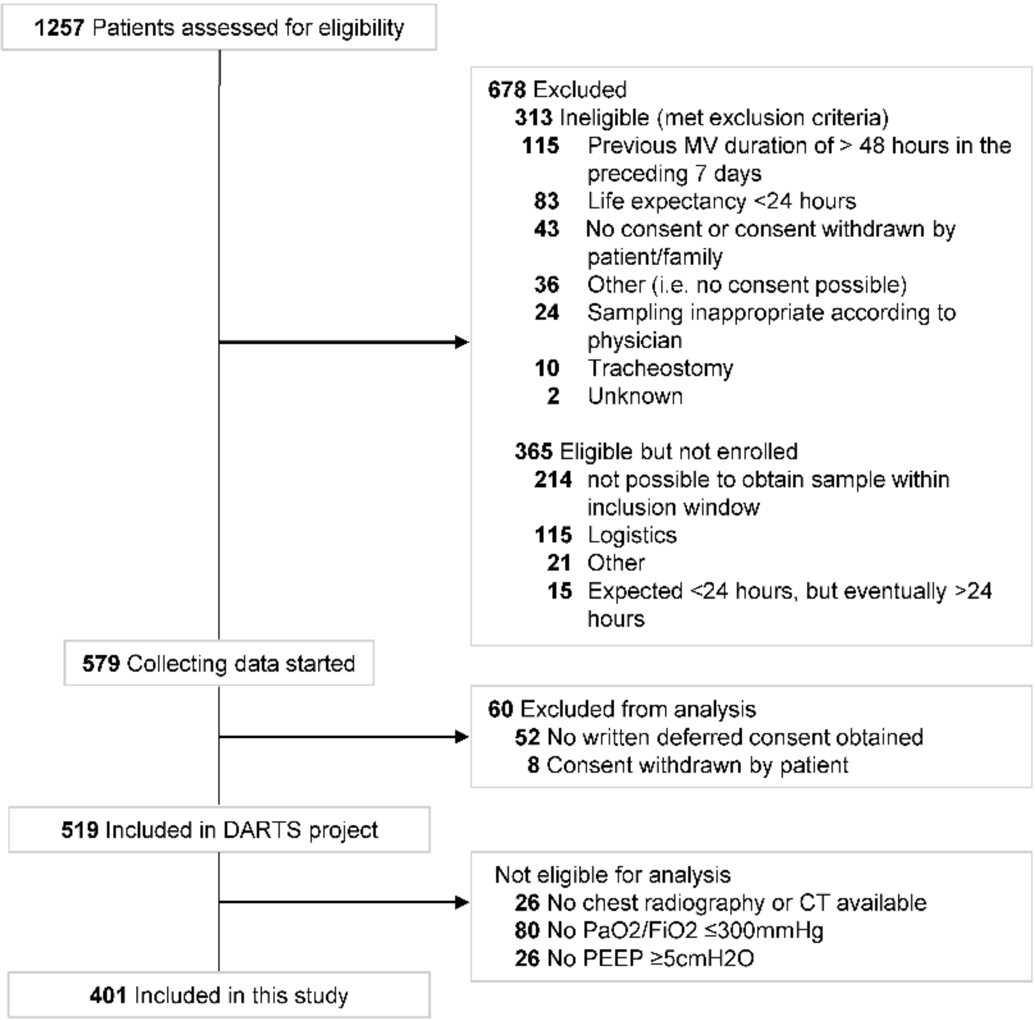

Flow of patients within the DARTS project and selected for this study.

Abbreviations: ARDS = acute respiratory distress syndrome, CT = computed tomography, FiO_2 = fraction of inspired oxygen, MV = mechanical ventilation, PaO_2 = partial pressure of oxygen, PEEP = positive end-expiratory pressure.

E-tables

E-table 1. Agreement for each imaging modality and situation.

Subset	*Modality*	*Situation*	*ICC (95%-CI)*	*N*	*Classification*
All available	CXR1	8-grade confidence scale	0.25 (0.07 – 0.41)	341	fair
	CXR1	dichotomous*	0.23 (0.09 – 0.35)	341	fair
	CXR2	8-grade confidence scale	0.24 (0.06 – 0.41)	99	fair
	CXR2	dichotomous	0.21 (0.04 – 0.39)	96	fair
	LUS1	8-grade confidence scale	0.49 (0.29 – 0.63)	350	moderate
	LUS2	8-grade confidence scale	0.53 (0.35 – 0.67)	193	moderate
Subset with CT available	CXR1	8-grade confidence scale	0.25 (0.06 – 0.42)	155	fair
	CT	8-grade confidence scale	0.49 (0.34 – 0.61)	202	moderate
	CXR1	dichotomous*	0.23 (0.09 – 0.36)	155	fair
	CT	dichotomous*	0.42 (0.28 – 0.54)	202	moderate

**dichotomous refers to the scoring of bilateral opacities on chest imaging compatible with ARDS.*

CI = confidence interval, CT = chest computed tomography, CXR1 = chest X-ray on day 1, CXR2 = chest X-ray on day 2, ICC = intra class correlation coefficient, LUS1 = lung ultrasound on day 1, LUS2 = lung ultrasound on day 2.

E-table 2. Difference in agreement between dichotomous assessment and 8 grade confidence scale

	Agreement and differences, per modality of each scoring method, ICC			
Modality	*ICC dichotomous**	*ICC 8-grade confidence scale*	*ICC difference [95%-CI]*	*N*
CXR1	0.23	0.25	0.022 [0.020, 0.023]	341
CXR2	NA	0.22	NA	74
CT	0.42	0.49	0.065 [0.063, 0.067]	202
LUS1	NA	0.50	NA	317
LUS2	NA	0.42	NA	74

**dichotomous refers to the scoring of bilateral opacities on chest imaging compatible with ARDS.*

CI = confidence interval, CT = chest computed tomography, CXR1 = chest X-ray on day 1, CXR2 = chest X-ray on day 2, ICC = intra class correlation coefficient, LUS1 = lung ultrasound on day 1, LUS2 = lung ultrasound on day 2.

E-table 3. Difference in agreement for imaging modalities and scoring methods.

	Difference in agreement between two scoring based on different imaging modalities, ICC [CI]		
Comparison between modalities	*Dichotomous**	*8-grade confidence scale*	*N*
CXR1 vs CT	0.20 [0.20, 0.21]	0.25 [0.24, 0.25]	155
CXR1 vs LUS1	NA	0.25 [0.25, 0.25]	317
CXR2 vs LUS2	NA	0.20 [0.19, 0.20]	74

**dichotomous refers to the scoring of bilateral opacities on chest imaging compatible with ARDS.*

CI = confidence interval, CT = chest computed tomography, CXR1 = chest X-ray on day 1, CXR2 = chest X-ray on day 2, ICC = intra class correlation coefficient, LUS1 = lung ultrasound on day 1, LUS2 = lung ultrasound on day 2.

E-table 4. Patient characteristics stratified per category.

	ARDS	likely ARDS	likely no ARDS	No ARDS	p-value
N	112	73	79	137	
Patient characteristic					
Age, years	62 (13)	64 (12)	62 (13)	63 (15)	0.70
Gender = Male	83 (74.1)	45 (61.6)	54 (68.4)	95 (69.3)	0.36
BMI	26.8 [23.7, 30.3]	27.3 [24.9, 30.2]	28.4 [24.7, 30.9]	25.5 [22.7, 29.5]	0.013
Admission characteristic					
Admission type					
Emergency surgical	3 (2.7)	12 (16.4)	10 (12.7)	28 (20.4)	<0.001
Medical	105 (93.8)	52 (71.2)	60 (75.9)	80 (58.4)	
Planned surgical	4 (3.6)	9 (12.3)	9 (11.4)	29 (21.2)	
ARDS severity					
mild	7 (6.2)	17 (23.3)	NA	NA	
moderate	61 (54.5)	36 (49.3)	NA	NA	
severe	44 (39.3)	20 (27.4)	NA	NA	
ARDS research team	95 (84.8)	27 (37.0)	4 (5.1)	14 (10.2)	
ARDS clinical team	12 (10.7)	4 (5.5)	1 (1.3)	3 (2.2)	
COVID-19	51 (45.5)	11 (15.1)	0 (0.0)	1 (0.7)	
Apache II	20 [13, 24]	20 [17, 25]	20 [15, 25]	21 [17, 26]	0.014
SOFA	8 [6, 11]	9 [7, 11]	9 [8, 11]	10 [8, 12]	0.003
LIPS	6.0 [4.5, 7.0]	5.5 [4.5, 7.5]	4.5 [3.0, 6.3]	5.0 [3.5, 7.0]	<0.001
Data at moment of first assessment					
MV duration at first assessment, hours	21 [11, 32]	21 [14, 26]	21 [15, 27]	22 [14, 30]	0.95
Compliance, mL/cmH2O	27.5 [20.9, 41.2]	33.0 [24.7, 43.3]	37.6 [26.3, 47.0]	33.3 [24.1, 50.7]	0.11
PaO2/FiO2, mmHg	112 [81, 143]	130 [98, 191]	168 [129, 234]	199 [143, 270]	<0.001
PEEP, cmH2O	10 [10, 12]	8 [7, 10]	8 [6, 9]	8 [5, 8]	<0.001

Imaging availability					
CXR1 available	94 (83.9)	64 (87.7)	71 (89.9)	117 (85.4)	0.66
CXR2 available	32 (28.6)	15 (20.5)	16 (20.3)	40 (29.2)	0.31
CT available	82 (73.2)	30 (41.1)	32 (40.5)	79 (57.7)	<0.001
Outcomes					
Hospital LOS, days	21 [12, 34]	18 [11, 27]	18 [9, 36]	21 [10, 32]	0.57
ICU LOS, days	9.0 [6.0, 19.0]	7.0 [3.0, 11.0]	6.0 [3.0, 10.8]	7.0 [3.0, 14.8]	0.001
ICU mortality	45 (40.2)	23 (31.5)	22 (27.8)	41 (29.9)	0.35
30d mortality	45 (40.2)	30 (41.1)	29 (36.7)	48 (35.0)	0.82

Data presented as no. (%) or median with interquartile range unless indicated otherwise. The PaO_2/FiO_2 is defined as the lowest PaO_2/FiO_2 in the 24h before day 1. ARDS = acute respiratory distress syndrome, BMI = body mass index, APACHE II = acute physiology and chronic health evaluation II, LIPS = lung injury prediction score, SOFA = sequential organ failure assessment, ICU = intensive care unit, LOS = length of stay, MV = mechanical ventilation, NA = not applicable, PEEP = positive end-expiratory pressure, FiO_2 = fraction of inspired oxygen, PaO_2 = partial pressure of oxygen.

E-table 5. Diagnostic characteristics for diagnosis of ARDS

	**Accuracy [95% CI]**	**sensitivity**	**specificity**	**PPV**	**NPV**
Clinical team	0.57 [0.52, 0.62]	0.98	0.09	0.56	0.80
Research team	0.80 [0.76, 0.84]	0.92	0.66	0.76	0.87
Expert 1	0.73 [0.68, 0.77]	0.98	0.43	0.67	0.94
Expert 2	0.86 [0.82, 0.89]	0.87	0.85	0.87	0.84
Expert 3	0.72 [0.67, 0.76]	0.53	0.95	0.92	0.63

Diagnostic characteristics for each group compared to expert panel classification.

CI = confidence interval, NPV = negative predictive value, PPV = positive predictive value.
